# Supplementary material for: Muscle fibroblasts and stem cells stimulate motor neurons in an age and exercise‐dependent manner
Source: Aging Cell. 2024 Nov 18;24(3):e14413. doi: 10.1111/acel.14413 (PMC11896526; doi:10.1111/acel.14413)
Supplement: Supplementary file 1 — Appendix S1. [file ACEL-24-e14413-s005.docx]

**Fig. S1.**

Differentially regulated human genes in Young vs Old after 1 day for FIB (blue) and MuSC

(red). Data are means (horizontal line) with individual values. Human n: Old: 12 (MuSC) and

8 (FIB). Young: 8 (MuSC) and 6 (FIB). Rat n: 7. Data were analyzed with DESeq2, see

bioinformatics section. Abbreviations: MuSC, muscle stem cell; FIB, muscle fibroblast.

**Table S1.**

Participants characteristics. Age (years), height (cm), weight (kg) and BMI (kg/m^2^) for all

participants. Data are means ± SD with ranges. BMI, body mass index.

**Movie S1.**

Differentiated primary human myotubes exhibiting synchronous contractions when exposed

to embryonic rat spinal cord explants.

**Movie S2.**

Differentiated primary human myotubes exhibiting synchronous contractions when exposed

to embryonic rat spinal cord explants.

**Movie S3.**

Differentiated primary human myotubes exhibiting synchronous contractions when exposed

to embryonic rat spinal cord explants.

**Data S1.**

Comparison of human MuSC versus FIB in co-culture with rat neuron cells. Positive log2-

fold difference = higher with MuSC. Relates to figure 1.

**Data S2.**

Comparison of Rat neuron cells co-cultured with either Human MuSC or FIB cells. Positive

log2-fold difference = higher with MuSC. Relate to figure 1 and 2.

**Data S3.**

Comparison of Rat neuron cells cultured with medium from either Human MuSC or FIB cells.

Positive log2-fold difference = higher with MuSC. Relates to figure 3.

**Fig. S1.**


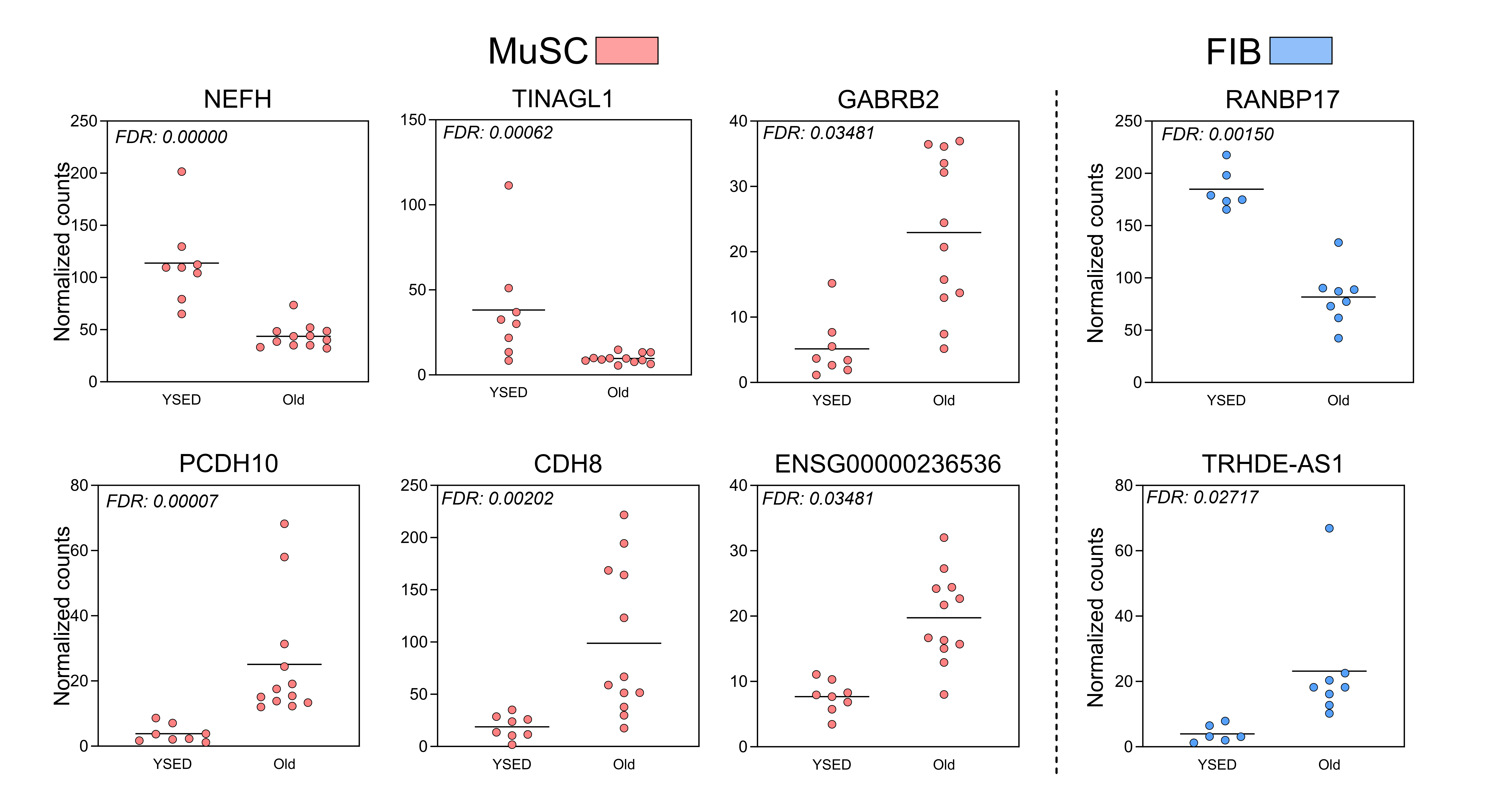


Differentially regulated human genes in Young vs Old after 1 day for FIB (blue) and MuSC

(red). Data are means (horizontal line) with individual values. Human n: Old: 12 (MuSC) and

8 (FIB). Young: 8 (MuSC) and 6 (FIB). Rat n: 7. Data were analyzed with DESeq2, see

bioinformatics section. Abbreviations: MuSC, muscle stem cell; FIB, muscle fibroblast.

**Table S1.**

|  | **Young (n=21)** | | | | | | | | | | | | **Old LLEX (n=7)** | | | | | | **Old SED (n=17)** | | | | | | | | | | | |
| --- | --- | --- | --- | --- | --- | --- | --- | --- | --- | --- | --- | --- | --- | --- | --- | --- | --- | --- | --- | --- | --- | --- | --- | --- | --- | --- | --- | --- | --- | --- |
|  | **Female (n=12)** | | | | | | **Male (n=9)** | | | | | | **Male (n=7)** | | | | | | **Female (n=11)** | | | | | | **Male (n=6)** | | | | | |
|  | *Mean* |  | *SD* | *min* |  | *maks* | *Mean* |  | *SD* | *min* |  | *maks* | *Mean* |  | *SD* | *min* |  | *maks* | *Mean* |  | *SD* | *min* |  | *maks* | *Mean* |  | *SD* | *min* |  | *maks* |
| Age | 23 | ± | 3 | 20 | - | 28 | 26 | ± | 5 | 20 | - | 34 | 72 | ± | 2 | 69 | - | 76 | 74 | ± | 3 | 71 | - | 78 | 73 | ± | 4 | 68 | - | 78 |
| Height (cm) | 168 | ± | 7 | 157 | - | 177 | 181 | ± | 8 | 169 | - | 191 | 176 | ± | 6 | 168 | - | 182 | 166 | ± | 3 | 162 | - | 169 | 176 | ± | 9 | 161 | - | 188 |
| Weight (kg) | 64 | ± | 8 | 53 | - | 75 | 81 | ± | 15 | 62 | - | 105 | 76 | ± | 9 | 70 | - | 94 | 69 | ± | 10 | 57 | - | 84 | 82 | ± | 6 | 71 | - | 88 |
| BMI (kg/m^2^) | 23 | ± | 2 | 19 | - | 26 | 25 | ± | 3 | 20 | - | 30 | 25 | ± | 3 | 22 | - | 30 | 25 | ± | 4 | 20 | - | 30 | 27 | ± | 3 | 23 | - | 32 |

Participants characteristics. Age (years), height (cm), weight (kg) and BMI (kg/m2) for all

participants. Data are means ± SD with ranges. BMI, body mass index.
